# Meta-analysis of uveal melanoma genome-wide association studies identifies novel risk loci and population effect size heterogeneity

**DOI:** 10.1016/j.xhgg.2025.100465

**Published:** 2025-06-09

**Authors:** Georgia Mies, Noah L. Tsao, Alexandre Houy, Sarah E. Coupland, Helen Kalirai, Asta Försti, Kari Hemminki, Hauke Thomsen, Marc-Henri Stern, Carol L. Shields, Scott M. Damrauer, Katheryn G. Ewens, Arupa Ganguly, Iain Mathieson

**Affiliations:** 1 Department of Genetics, University of Pennsylvania Perelman School of Medicine, Philadelphia, PA, USA; 2 Department of Surgery, University of Pennsylvania Perelman School of Medicine, Philadelphia, PA, USA; 3 INSERM U1339/CNRS UMR3666, DNA Repair and Uveal Melanoma (D.R.U.M.) Team, Institut Curie, PSL Research University, Paris, France; 4 Liverpool Clinical Laboratories, Liverpool University Hospitals Foundation Trust, Liverpool, UK; 5 Liverpool Ocular Oncology Research Group, Department of Eye and Vision Sciences, University of Liverpool, Liverpool, UK; 6 Hopp Children’s Cancer Center (KiTZ), Heidelberg, Germany; 7 Division of Pediatric Neurooncology, German Cancer Research Center (DKFZ), German Cancer Consortium (DKTK), Heidelberg, Germany; 8 Biomedical Center, Faculty of Medicine in Pilsen, Charles University, Pilsen, Czech Republic; 9 Division of Cancer Epidemiology, German Cancer Research Center (DKFZ), Heidelberg, Germany; 10 MSB Medical School Berlin, Berlin, Germany; 11 Ocular Oncology Service, Wills Eye Hospital, Thomas Jefferson University, Philadelphia, PA, USA; 12 Department of Surgery, Corporal Michael Crescenz VA Medical Center, Philadelphia, PA, USA

**Keywords:** uveal melanoma, genome-wide association study, eye pigmentation

## Abstract

Uveal melanoma (UM) is a rare but frequently metastasizing cancer. Genome-wide association studies have identified three common genome-wide significant germline risk loci. Here, we perform a genome-wide association study on 401 new cases and conduct a meta-analysis with three independent previously published cohorts for a total sample size of 2,426 cases. We confirm the three previously identified risk loci and identify four additional genome-wide significant loci. We find that eye pigmentation-decreasing variants are systematically associated with increased UM risk and that selection for lighter pigmentation in the past 5,000 years explains about 73% of the difference in UM incidence between Northern and Southern Europe. We find evidence of effect size heterogeneity at significant loci across cohorts, in particular, a weaker association between eye pigmentation and UM in a Finnish cohort. Finally, we confirm differential effect sizes between uveal melanoma cases with and without loss of chromosome 3, the major determinant of metastatic risk. Our study identifies novel germline risk factors for UM and highlights genetic and environmental heterogeneity in its etiology.

## Teaser

Mies and colleagues perform a meta-analysis of genome-wide association studies for uveal melanoma (UM), identifying novel loci associated with UM risk. They find that light eye pigmentation is associated with increased UM risk and that some variants may have differential effects on risk of metastasis.

## Introduction

Uveal melanoma (UM) is a rare cancer of which approximately 50% of cases develop metastatic disease, most commonly to the liver. While 90% of UM cases develop in the choroid, primary tumors can also occur in the ciliary body and the iris.^1^ Risk factors include age, light skin and iris color, Northern European ancestry, multiple skin naevi, nevus of Ota, and a family history of UM or cutaneous melanoma. Within Europe, incidence rates of UM range from around 10 cases per million people per year in Scandinavia to around 3 in Southern Europe.^2^ Common symptoms include blurred or distorted vision, vision loss, and changes in iris color (acquired heterotopia).^3^ Due to its metastatic potential, UM has a high 5-year mortality rate of 30%.^4^ Understanding genetic and environmental factors that contribute to UM risk could help improve early screening, detection, and prevention strategies as well as identify potential therapeutic targets.

Somatic driver mutations in UM primarily occur within the *GNA* gene family, particularly in *GNAQ* and *GNA11*.^5^ Germline risk factors include rare but highly penetrant *BAP1* loss-of-function mutations^6^ and common variants at three loci identified through genome-wide association studies (GWASs).^7^ Genes at two of these loci, *HERC2/OCA2* and *IRF4*, are involved in pigmentation,^8^^,^^9^^,^^10^^,^^11^ while the third, *CLPTM1L/TERT*, is a known cancer driver.^12^ In particular, homozygosity for the *HERC2* risk-increasing allele is the primary determinant of blue eye color, which is a risk factor for UM.^13^^,^^14^ Despite this, and in contrast to cutaneous melanoma, UMs do not show signatures of UV mutational damage.^5^ Instead, other non-UV wavelengths may initiate the cancer.^15^

UM clinical and molecular subtypes are determined by tumor location within the eye and chromosomal makeup.^16^ A key factor influencing metastatic potential is the status of chromosome 3. The loss of one copy of chromosome 3 (monosomy 3 [M3]) significantly increases the risk of developing metastatic disease compared to tumors with two copies (disomy 3 [D3]); M3 cases are 10 times more likely to metastasize than D3 cases, and metastasis is typically fatal within 4–5 years.^17^ A previous GWAS identified *HERC2* as exclusively associated with the M3 subtype and *IRF4* with D3.^7^

To better understand the environmental and genetic basis of UM susceptibility, we conducted a GWAS and analyzed the results together with existing GWAS results for UM and eye pigmentation. We aimed to identify germline risk factors and test whether variants associated with light eye color are, in general, associated with increased UM risk. Since both pigmentation levels and solar radiation exposure vary globally, we hypothesized that the effects of UM risk loci may differ between populations. Thus, we aimed to identify loci that exhibit effect size heterogeneity across cohorts. Finally, since metastatic potential is a key determinant of clinical outcomes, we aimed to identify loci with differential effects on M3 and D3 cases.

## Subjects and methods

### Wills Eye Hospital GWAS

#### Study populations

We recruited 409 UM cases via the UPenn Genetic Diagnostic Laboratory from patients receiving treatment at the Wills Eye Hospital (Philadelphia, PA, USA). We collected blood samples and patients’ sex, age, metastasis status, chromosome 3 status, tumor stage, tumor location, and eye color. We also selected 937 controls originally recruited as part of the Penn Medicine Biobank.^18^ This study was approved by the Perelman School of Medicine institutional review board (protocol 807701, Biomarkers for Uveal Melanoma).

#### Genotyping, imputation, and quality control

We genotyped cases and controls at 826,804 variants using the Affymetrix Axiom Precision Medicine Research Array and performed quality control, removing individuals with sex mismatch or high missingness (>5%), and SNPs with a genotype call rate <95%. We merged these with data from the 1000 Genomes Project^19^ and performed principal-component analysis (PCA) on cases and controls. Most individuals fell within the European cluster on the PCA (Figure S1), and we removed 8 cases and 164 controls as ancestry outliers based on the first two principal components. We filtered SNPs on Hardy-Weinberg equilibrium (HWE; *p* < 10^−6^), SNP missingness (<95%), and a minor-allele frequency (MAF) cutoff 0.01 and removed 11 controls due to individual missingness (>5%) using PLINK (v.1.90b5.4),^20^ leaving a final count of 762 controls and 401 cases.

We imputed cases and controls together on the Michigan Imputation Server^21^ using the GRCh37 1000 Genomes reference panel.^19^ We prepared samples for imputation using McCarthy Group Tools with EUR population options (https://www.chg.ox.ac.uk/∼wrayner/tools/). Finally, we applied post-imputation quality control filters of removing monomorphic sites, SNP missingness of 0.05, HWE (*p* < 10^−6^), individual missingness of 0.05 (0 individuals removed), and a MAF cutoff of 0.01 using PLINK and BCFtools.^22^ We manually restored SNPs that were identified in significant peaks by Mobuchon et al.^7^ summary statistics that were removed due to not passing the HWE cutoff (*n* = 21). This left a final count of 5,966,774 SNPs.

#### Statistical analyses

We ran the GWAS with GCTA v.1.93.2b using the fastGWA generalized linear mixed model with sex and the first three principal components as covariates).^23^ We also ran a GWAS separately for M3 (*n* = 165) and D3 cases (*n* = 149) and calculated *Z* scores for the difference between the two: $Z=\frac{{\hat{\beta }}_{M3}-{\hat{\beta }}_{D3}}{\sqrt{\sigma_{M3}^{2}+\sigma_{D3}^{2}}}$, where ${\hat{\beta }}_{M3/D3}$ and $\sigma_{M3/D3}^{2}$ represent the estimated effect size and standard error for the M3/D3 GWAS, respectively.

To estimate whether *HERC2* has a recessive effect on UM risk as it does on blue eye color, we used logistic regression with the model where case/control status Y∼genotype+dominanceencoding+sex+PCs1:3. The dominance encoding is defined by mapping genotype counts [0,1,2] to [-p/(1-p), 1, -(1-p)/p], where p is the MAF.^24^

### GWAS meta-analysis

We conducted a meta-analysis using METAL^25^ with summary statistics from the Wills Eye Hospital GWAS described above, Mobuchon et al.,^7^ Thomsen et al.,^26^ and FinnGen (release 11, endpoint CD2_UVEAMELANOMA_EXALLC).^27^ We lifted over FinnGen summary statistics from hg38 to hg19 using liftOver.^28^ We removed variants private to a single study, leaving a combined total of 7,820,481 SNPs. We applied genomic control to all datasets before meta-analysis and again on the meta-analysis results. We computed heterogeneity of significant locus (*n* = 7) effect sizes across input GWAS summary statistics using the “analyze heterogeneity” command of METAL and defined significant *p* values at a threshold of *p* <0.05/7 = 0.007.

We identified genome-wide significant hits at *p* < 5 × 10^−8^ and plotted local association plots using the LocusZoom online interface.^29^ To test for replication of hits from each study, we ran meta-analyses with each dataset individually excluded from the analysis and then queried significant and nominally significant SNPs (*p* < 10^−6^, *n* = 45) from the excluded dataset in the meta-analysis results. We defined replicated SNPs as those with a *p* value less than 0.05/45 = 0.0011.

### Genetic correlation between UM and eye pigmentation

We computed correlations between UM and eye pigmentation using the UM meta-analysis results and a published eye pigmentation GWAS.^30^ For eye pigmentation, we used 52 reported genome-wide significant SNPs and computed the Pearson’s correlation between the effect of the pigmentation-increasing allele on eye color and the UM beta weighted by the standard errors of both estimates. We assessed significance of the correlation by permuting effect sizes for one million replicates.^30^^,^^31^ We performed two-sample Mendelian randomization using the R package MendelianRandomization v.0.9.0^32^ with eye pigmentation betas as the exposure variable and UM meta-analysis betas as the outcome variable. Additionally, we applied Deming regression of UM meta-analysis betas (with and without FinnGen) on eye pigmentation betas, incorporating the standard errors of each GWAS estimate.

Similarly, we computed the correlation between eye pigmentation betas and the Wills Eye Hospital M3 and D3 GWAS betas. We calculated *Z* scores for the difference between the two correlations using the same calculation used for M3 and D3 difference in effect size estimate, with ${\hat{\beta }}_{M3/D3}$ and $\sigma_{M3/D3}^{2}$ representing the correlation between the betas and standard error for the correlation for the M3/D3 GWAS, respectively.

### Estimating the effects of changes in eye pigmentation allele frequencies on UM incidence

To estimate how much differences in pigmentation allele frequencies across European populations contribute to the difference in risk, we calculate the difference in allele frequency $\delta f_{i}\text{,}$ for each SNP *i* and estimate the odds ratio due to this change in allele frequency using the equation ${R=e}^{\sum 2\beta_{i}\delta f_{i}}$, where $\beta_{i}$ is the effect size of SNP *i* estimated from logistic regression. Then, we calculate the difference (*p-q*) for Northern vs. Southern Europe, Finland vs. Southern Europe, and Finland vs. Northern Europe using samples from the 1000 Genomes Project (CEU and GBR, TSI and IBS, and FIN)^19^ by substituting the Southern Europe incidence of UM or Northern Europe for the third analysis for *p* and solving for incidence of the other population (*q*) in the equation $\frac{p\left(1-q\right)}{q\left(1-p\right)}=R$ (i.e., the definition of the odds ratio).

To estimate the impact of natural selection on UM risk, we used ancient DNA from 686 individuals^33^^,^^34^^,^^35^^,^^36^^,^^37^^,^^38^ spanning the last 5,000 years in Great Britain (data processing and imputation are described by Poyraz et al.^39^). We estimated the change in allele frequency, $\delta f_{i}$, for each SNP *i* by applying linear regression to genotype data over time and then multiplied the regression slope by 5,000. We estimated the odds ratio due to this change in allele frequency and the past incidence (*q*) of UM using the known current incidence (*p*) and the above equations.

### Ethics statement

The Wills Eye Hospital GWAS was approved by the institutional review board of the Perelman School of Medicine, University of Pennsylvania; other studies were approved as described in the original publications.

## Results

### Seven genome-wide significant associations

**Figure 1:**
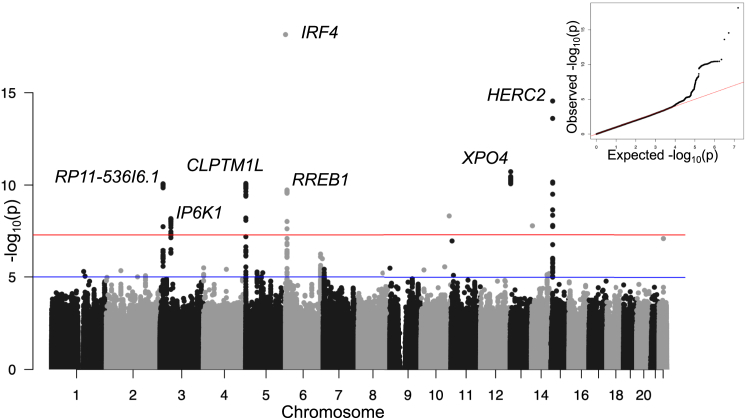
Manhattan plot of the meta-analysis of four independent UM GWASs Inset: quantile-quantile plot of the meta-analysis with genomic control applied. The nearest gene to the lead SNP of the seven genome-wide significant peaks is annotated. Red and blue horizontal lines denote genome-wide and nominal significance thresholds.

The Wills Eye Hospital GWAS did not identify any genome-wide significant hits and had 17 nominally significant hits (*p* < 10^−6^) (Table S1). In a meta-analysis of the Wills Eye Hospital (*n* = 401 cases), Mobuchon et al.^7^ (*n* = 1,142 cases) , Thomsen et al.^26^ (*n* = 590 cases), and FinnGen (*n* = 293 cases)^7^^,^^26^^,^^27^ studies (Table 1), we identified seven genome-wide significant loci (*p* < 5 × 10^−8^; Figure 1; Table S2). These include three genome-wide significant loci reported previously by Mobuchon et al. at loci encoding the pigmentation genes *HERC2/OCA2* and *IRF4,* and a cancer driver gene at the *CLPTM1L/TERT* locus*.* The four additional genome-wide significant loci include two loci reported as nominally significant by Thomsen et al., *RP11-536I6.1* and *RREB1*; a locus reported by Thomsen et al. as associated with subtype epithelioid cell UM at *XPO4*; and one novel peak not reported previously at *IP6K1*. Two additional SNPs on chromosomes 10 and 14 (rs1278278 and rs12889516) reached genome-wide significance in the meta-analysis. However, these two SNPs show inconsistent direction of effect across datasets and are not supported by additional SNPs in linkage disequilibrium (LD) (Figure S2), so we excluded them.

**Table 1:** Contributing GWASs

Study (year)	Country; ancestry	Cases/controls	Significant; nominal hits	Hits replicating
Wills Eye Hospital (2024)	USA; European	401/762	0; 17	0; 0
Mobuchon et al. (2022)^7^	France; non-Finnish European	1,142/882	3; 8	3; 0
FinnGen (2023)^27^	Finland; Finnish	293/345,118	1; 8	0; 0
Thomsen et al. (2020)^26^	UK; Northwest European	590/5,199	0; 11	0; 2

Shown are the four GWASs performed on independent datasets, with corresponding country of collection and ancestry filtering of cases and controls, number of cases and controls in each study, number of independent significant hits (*p* < 5 × 10^−8^) identified in the GWAS and the number of nominal hits (*p* < 10^−6^), and, last, the number of hits (significant and nominal) from each GWAS that are replicated in the corresponding meta-analysis, excluding the target GWAS summary statistics.

**Figure 2:**
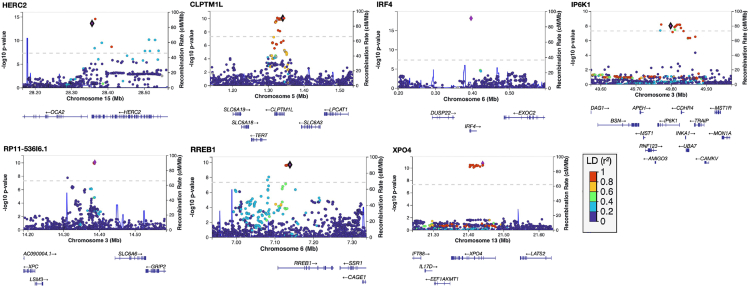
Seven genome-wide significant hits Shown are LocusZoom plots of the meta-analysis results showing−log_10_*p* values (*y* axis) and chromosome and base pair position (*x* axis). Nearby genes are annotated below the plots. LD is based on European populations.

Three genome-wide significant associations have been reported previously by Mobuchon et al.^7^ The most statistically significant was at *IRF4*, which encodes a transcription factor involved in regulating production of melanin and is also involved in immune response.^40^^,^^41^ The association is driven by a single SNP (rs12203592, *p* = 7.10 × 10^−19^; Figure 2) but is consistent across studies and with the effect of this SNP on pigmentation.^30^ The second lead SNP was rs12913832 in *HERC2* (*p* = 2.84 × 10^−15^). This intronic SNP is the major determinant of blue eye color through regulation of nearby *OCA2*.^42^^,^^43^^,^^44^ The third recapitulated hit, at the *CLPTM1L/TERT* locus*,* has been implicated in other cancers, including lung cancer and melanoma (rs421284, *p* = 8.24 × 10^−11^).^12^

Associations at *RP11-536I6.1* and *RREB1* have been identified previously as nominally significant by Thomsen et al.^26^ The *RP11-536I6.1* locus (rs11707457, *p* = 8.57 × 10^−11^) contains several genes, including *XPC* and *SLC6A6*. *XPC* encodes a DNA damage recognition protein, with mutations linked to cutaneous melanoma, particularly in individuals with UV-induced damage.^45^^,^^46^ Another candidate gene, the taurine transporter *SLC6A6*, has been linked to early retinal degeneration.^47^ *RREB1* (rs551143, *p* = 1.84 × 10^−10^) codes for a zinc-finger transcription factor involved in cell proliferation and DNA damage repair and has been identified previously in GWASs of cutaneous melanoma, bladder cancer, and age-related macular degeneration.^31^^,^^48^^,^^49^^,^^50^ SNPs within *XPO4* have been identified in GWASs of cutaneous melanoma as well as other non-cancer related traits (*p* = 1.9 × 10^−11^).^51^ *XPO4* was nominally associated with the “epithelioid tumor cell type” subtype by Thomsen et al.^26^ It is involved in cellular transport and has been identified as a potential tumor suppressor gene in liver cancer models.^52^ Although several genes at the *IP6K1* locus (*p* = 6.57 × 10^−9^) have been linked to various cancers, the causal gene is unknown.^53^ While the genes identified in the meta-analysis show promising associations with UM, additional functional studies are necessary to validate their biological roles in UM development and progression.

### Heterogeneity in effects across studies

**Figure 3:**
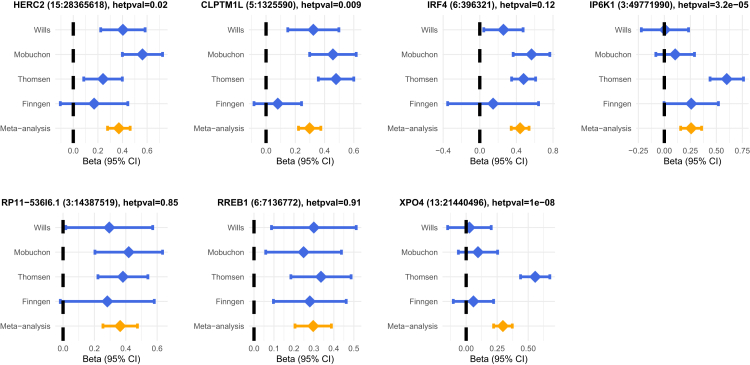
Effect size heterogeneity Shown are forest plots of effect sizes as beta for each GWAS study (denoted Wills, Mobuchon, Thomsen, and FinnGen) and meta-analysis (orange) with 95% CI on the *x* axis for each of the seven genome-wide significant peaks’ lead SNP. For each lead SNP, the title contains the closest gene, chromosome, and base pair position (hg19) and the heterogeneity *p* value across the four GWASs in the meta-analysis.

We evaluated the differences in effect sizes across the four GWASs included in the meta-analysis (Figure 3). *HERC2* and *CLPTM1L* have nominally significant heterogeneity across studies (*p* = 0.02, 0.009), largely driven by smaller effect sizes in FinnGen. In fact, the 95% confidence intervals for FinnGen effect sizes for *HERC2*, *IRF4*, and *CLPTM1L* all overlap 0. We hypothesize that this reflects a distinct pattern of environmental exposure in Finland (see discussion), although we cannot rule out differences in population stratification or case ascertainment that might shrink effects in FinnGen or inflate them in other cohorts. *XPO4* and *IP6K1* have highly significant differences in effect (*p* = 1 × 10^−8^, 3.2 × 10^−5^), both reflecting large effects in Thomsen et al. and smaller or zero effects in other studies. Associations at *RP11-53616* and *RREB1* are highly consistent across studies.

### Failure to replicate nominally significant hits

Previous studies reported multiple “nominally” significant associations at a level below genome-wide significance (Mobuchon et al., 8 hits at *p* < 10^−6^; Thomsen et al., 11 hits at *p* < 10^−6^). We also identified 17 loci in the Wills Eye Hospital study and 8 in FinnGen at *p* < 10^−6^. For each of these, we ran a meta-analysis excluding the target GWAS to test for replication. Two of these loci, at *RP11-536I6.1* and *RREB1*, both from Thomsen et al. (which were genome-wide significant in the full meta-analysis) replicate at a *p* value threshold of 0.0011 with the original study excluded (Table S3). All other nominal associations failed to replicate (Table S4).

### Differences in effect sizes in M3 and D3 cases in the Wills Eye Hospital GWAS

**Figure 4:**
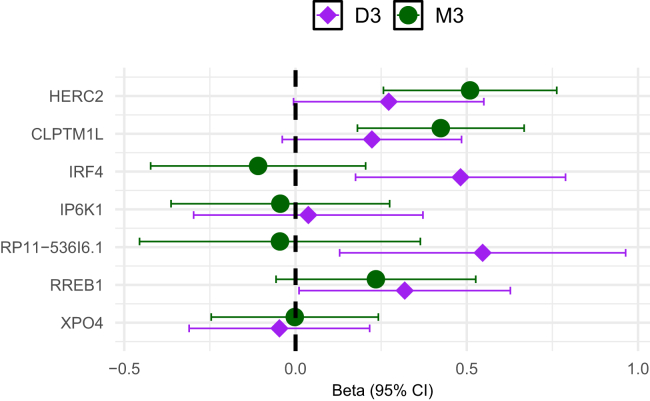
Differential effects on D3 and M3 risk Shown is a comparison of Wills Eye Hospital GWAS UM betas from cases with disomy 3 (D3) or monosomy 3 (M3). The *y* axis shows the gene name of the seven genome-wide significant hits identified in the meta-analysis results. The *x* axis shows the 95% CI for betas from each GWAS.

We compared effect sizes between cases with M3 and D3 subtypes in the Wills Eye Hospital GWAS (Figure 4). *IRF4* effect sizes differ significantly between M3 and D3 cases (*p* = 0.004, M3 *p* = 0.49, D3 *p* = 0.002), consistent with being exclusively associated with D3 as reported by Mobuchon et al.^7^ Although effect sizes are not significantly different between M3 and D3 for *HERC2*, only M3 is individually significant (*p* = 0.107, M3 *p* = 7.8 × 10^−5^, D3 *p* = 0.05), again consistent with the result of Mobuchon et al. that *HERC2* is exclusively associated with M3 or at least has a much larger effect.^7^ One possibility is that these differences reflect pleiotropic effects of *HERC2* or *IRF4* beyond their effect on pigmentation. The *HERC2* locus is associated with blue eye color through regulation of *OCA2*, but *HERC2* is also directly involved in DNA repair.^44^^,^^54^ Similarly, *IRF4* directly affects pigmentation but also has an important role in immune regulation.^41^ *RP11-536I6.1* effect sizes are not significantly different but show a qualitatively similar pattern to *IRF4* (*p* = 0.024, M3 *p* = 0.828, D3 *p* = 0.010) potentially consistent with a larger effect on D3 than M3. *CLPTM1L*, *IP6K1*, *XPO4*, and *RREB1* did not show significant differences between M3 and D3 GWAS effect sizes.

### Effect of eye pigmentation on UM risk

**Figure 5:**
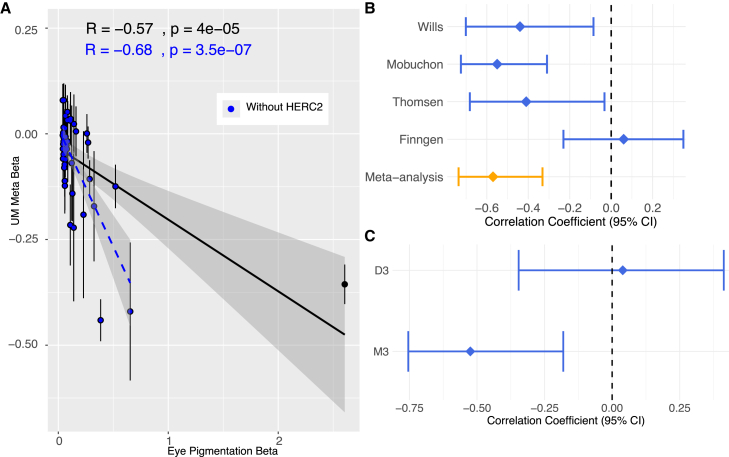
Relationship between eye pigmentation and UM risk (A) Correlation of UM meta-analysis GWAS and eye pigmentation GWAS effect sizes, reported both with and without *HERC2* included in the analysis. Note that the error bars for eye pigmentation betas are too small to be seen on this plot. (B) Correlation between UM and eye pigmentation GWAS effect sizes for each of the GWASs analyzed in the meta-analysis. (C) Correlation between eye pigmentation effect sizes and, separately, Wills Eye Hospital M3 and D3 effect sizes.

Light eye color is a risk factor for UM, and two genome-wide significant hits have been identified in pigmentation genes. However, eye color covaries with both genetic ancestry and environment, making it difficult to fully establish the causal relationship between eye pigmentation and UM risk. We investigated this relationship by estimating the correlation between eye color GWAS effect sizes and UM meta-analysis effect sizes for 52 independent SNPs associated with eye color.^30^ The correlation between eye pigmentation and UM betas was −0.57 (permutation *p* = 0.0025; −0.68, *p* = 6 × 10^−5^ without *HERC2*; Figures 5A and S3). This correlation is consistent across all studies except FinnGen, where the correlation is not significantly different from zero (Figure 5B). Deming regression of UM betas on eye pigmentation betas (considering the standard errors of the estimates) estimates a slope of −0.16 (95% confidence interval [CI]: [−0.57, 0.25], [−0.70, −0.04] without *HERC2*) and −0.18 (95% CI: [−0.70, 0.33], [−0.81, −0.08] without *HERC2*). Finally, two-sample Mendelian randomization with eye pigmentation betas as the exposure variable and UM betas as the outcome variables supports a causal effect of eye pigmentation on UM risk (beta = −0.162, CI: [−0.214, −0.110] *p* < 0.001). Although this causal relationship is plausible, we caution that these analyses could still be confounded by factors such as pleiotropy (e.g., as the *HERC2* and *IRF4* associations described above) and correlated environmental factors.

The effect of rs12913832 in *HERC2* on blue vs. brown eye color is recessive.^24^^,^^43^ However, its effect on skin and hair pigmentation is largely additive,^24^ and we find that its effect on UM risk is also largely additive in the Wills cohort (additive effect, *p* = 2.01 × 10^−6^; dominant/recessive effect, *p* = 0.20). Therefore, while blue eye color is a risk factor for UM, rs12913832 heterozygotes also have increased risk compared to homozygotes for the ancestral allele, likely due to lighter (but still brown-appearing) pigmentation.

Finally, we tested the correlation between eye pigmentation betas and, separately, M3 and D3 GWAS betas in the Wills Eye Hospital GWAS (Figure 5C). The correlation with M3 was significantly less than zero, whereas the correlation with D3 was not significantly different than zero (*p* = 0.01, M3 = −0.524; D3 = 0.039). This suggests that light eye pigmentation may be specifically a risk factor for M3, which would be consistent with the larger effect of *HERC2* on M3, although inconsistent with the larger effect of *IRF4* on D3.

### Effects of differences in pigmentation allele frequencies across populations on UM risk

Pigmentation is one of the most strongly selected traits in humans, and there has been ongoing selection for lighter pigmentation in Europe even in the past few thousand years,^55^ likely due to the advantage of lighter pigmentation in vitamin D biosynthesis in high-latitude regions.^56^ This may have contributed to the increased risk of UM in Northern Europe, where UM incidence is about twice that of Southern Europe (6 vs. 3 per million people per year).^57^ We estimate that differences in pigmentation allele frequencies between Northern and Southern Europe (specifically, Britain vs. Spain and Italy) can explain a large fraction of this difference (2.19 cases per million people per year, or 73% of the difference). Furthermore, all of this difference can be explained by allele frequency changes over the last 5,000 years, with *HERC2* making the largest contribution. Incidence of UM in Scandinavia, including Finland, is even higher than in other parts of Northern Europe (approximately 8 cases per million people per year in Finland),^2^ but our analysis suggest that this difference cannot be explained by differences in pigmentation allele frequencies (which would actually predict 0.31% lower incidence in Finland vs. Britain). This, combined with the observation that rates of UM in Scandinavia have increased substantially over the past 60 years,^58^^,^^59^ suggests that high rates of UM in Scandinavia reflect differences in diagnosis or environment rather than differences in genetic susceptibility.

## Discussion

We replicate three established UM risk alleles and identify four new genome-wide significant loci. Of these four, two are driven by a single study, and two appear to be very consistent across studies. Loci with known function are involved in pigmentation or are cancer driver genes. We observe heterogeneity of effect sizes across studies, and six of the FinnGen effect size confidence intervals overlap 0.

The largest effects are at *HERC2* and *IRF4*; pigmentation-related genes that have undergone significant selection in European populations over the last 10,000 years.^8^ One concern is that selection can induce population stratification that would not be controlled by genome-wide principal components; we note that SNPs from both *HERC2* and *IRF4* were originally removed in HWE filtering in the Wills Eye Hospital GWAS and were restored manually. Despite this, the plausible mechanisms of action at these genes mean that the associations are likely real, although effect sizes might be mis-estimated.

We find *IRF4* to be associated with D3 cases, which correspond to a decreased metastatic risk. In contrast, *HERC2*, although not statistically significant, is consistent with the findings of Mobuchon et al. (M3 cases, increased metastatic risk). Additionally, effect sizes for lighter eye pigmentation were correlated with UM risk for M3 but not D3. This supports the association of *HERC2* with M3 cases and suggests that lighter eye color may be more strongly linked to M3 than D3 risk. Previous studies have found that light eye color is negatively correlated with survival among M3 cases,^60^ but it remains to be seen whether these two observations are related. *RP11-536I6.1* had some evidence of difference in M3 and D3 effect sizes, although it was not significant after correcting for multiple testing (*p* = 0.024). Larger studies of the differences between M3 and D3 cases may reveal genome-wide significant hits that could predict individual susceptibility to metastasis.

We find evidence of a causal effect of eye pigmentation on UM risk and estimate that a substantial fraction of the absolute difference in risk between Northern and Southern Europe is driven by differences in eye pigmentation allele frequency. Nonetheless, this still leaves some difference in risk to be explained either by other genetic factors or by environment. Surprisingly, we find that the effect of eye pigmentation on UM risk is smaller or zero in Finland. One explanation might be that, due to lower levels of incident solar radiation, light pigmentation has less effect on risk. But in that case, we would expect lower overall incidence in Finland, which is the opposite of what is observed. A more likely explanation is that much of the risk of UM in Finland is due to environmental risk factors that do not interact with pigmentation. This would be consistent with the observation that higher UM incidence in Scandinavia cannot be explained by differences in pigmentation allele frequencies and likely reflects environmental or behavioral differences.^58^

Finally, while UM is predominantly diagnosed in individuals of European ancestry, it can also affect non-European populations, with UM occurring in all parts of the world.^2^ These groups are underrepresented in current GWASs, and expanding studies to include additional ancestries could identify additional risk variants and environmental factors and clarify the relationship between pigmentation and UM risk.

## Data and code availability

Summary statistics for the meta-analysis are available at the NHGRI-EBI GWAS catalog (www.ebi.ac.uk/gwas) via accession number GCST90568461.

## Acknowledgments

This work was supported in part by the 10.13039/100000057National Institute of General Medical Sciences R35GM133708 (to I.M.). K.H. was supported by the SALVAGE project, registration number CZ.02.01.01/00/22_008/0004644. We thank Quincy Blubaugh, B.S., for assistance with genotyping.

## Declaration of interests

S.M.D. receives research support from Novo Nordisk and consulting fees from Tourmaline Bio.

## Supplemental information

Document S1. Figures S1–S3 and Tables S1–S4

Document S2. Article plus supplemental information
