# Supplementary material for: Meta-analysis of uveal melanoma genome-wide association studies identifies novel risk loci and population effect size heterogeneity
Source: HGG Adv. 2025 Jun 9;6(3):100465. doi: 10.1016/j.xhgg.2025.100465 (PMC12226357; doi:10.1016/j.xhgg.2025.100465)
Supplement: Document S1. Figures S1–S3 and Tables S1–S4 [file mmc1.pdf]

HGGA, Volume *6*

Supplemental information

Meta-analysis of uveal melanoma genome-wide association studies identifies novel risk loci and population effect size heterogeneity

Georgia Mies, Noah L. Tsao, Alexandre Houy, Sarah E. Coupland, Helen Kalirai, Asta Försti, Kari Hemminki, Hauke Thomsen, Marc-Henri Stern, Carol L. Shields, Scott M. Damrauer, Katheryn G. Ewens, Arupa Ganguly, and Iain Mathieson

Supplementary Tables and Figures

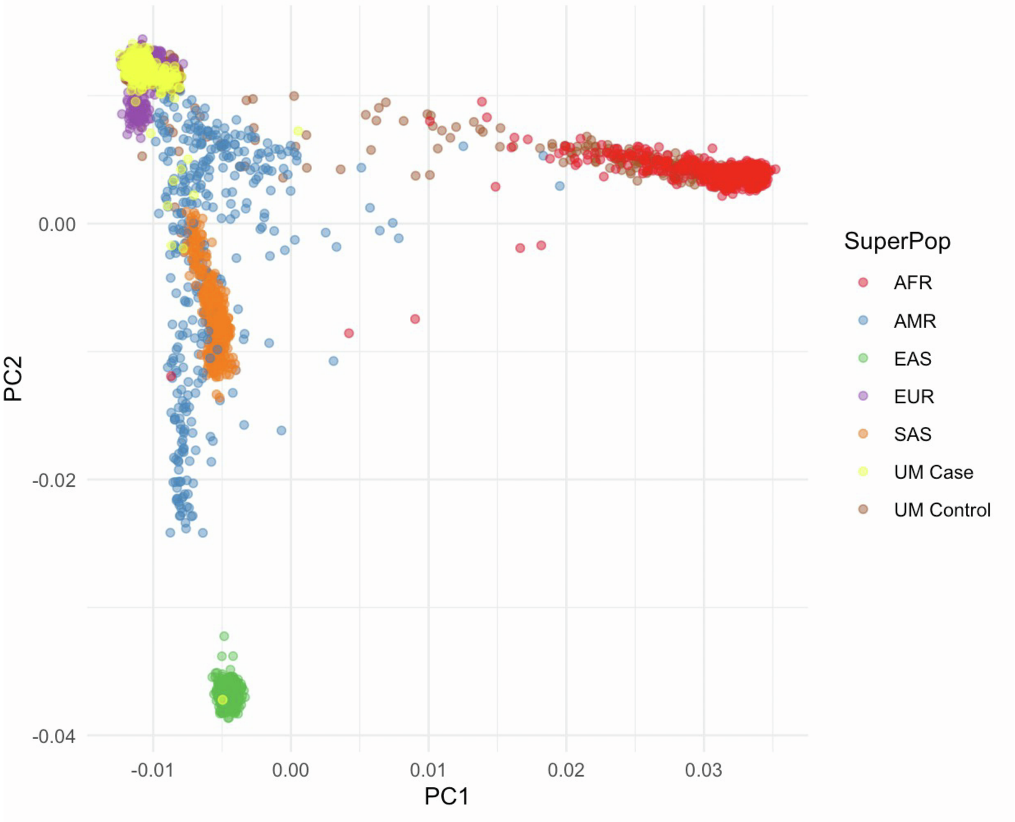

Figure S1. PCA of Wills Eye Hospital GWAS cases, controls, and 1000 Genomes populations (AFR, AMR, EAS, EUR, SAS).

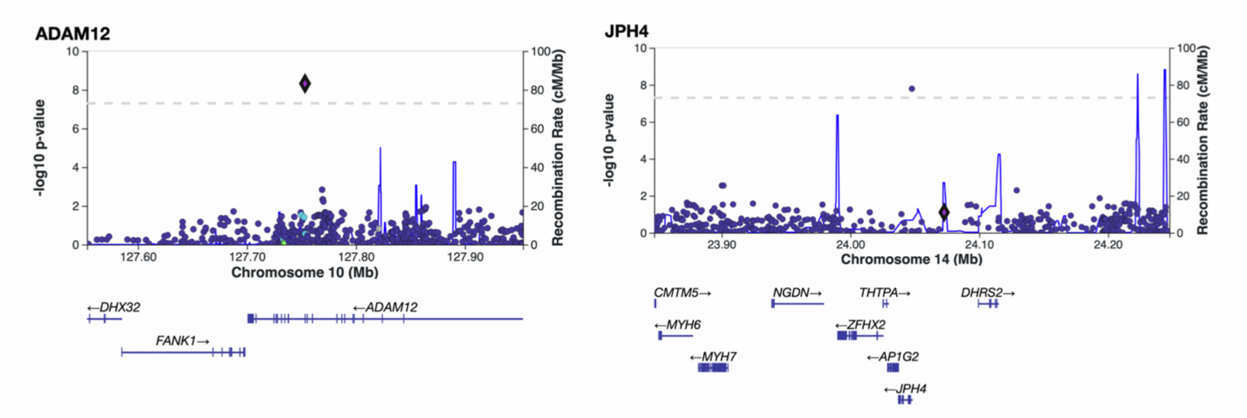

Figure S2. Locus Zoom plots of the two additional SNPs on chromosome 10 and 14 (rs1278278 and rs12889516) that reached genome-wide significance in the meta-analysis but are not supported by additional SNPs in LD.

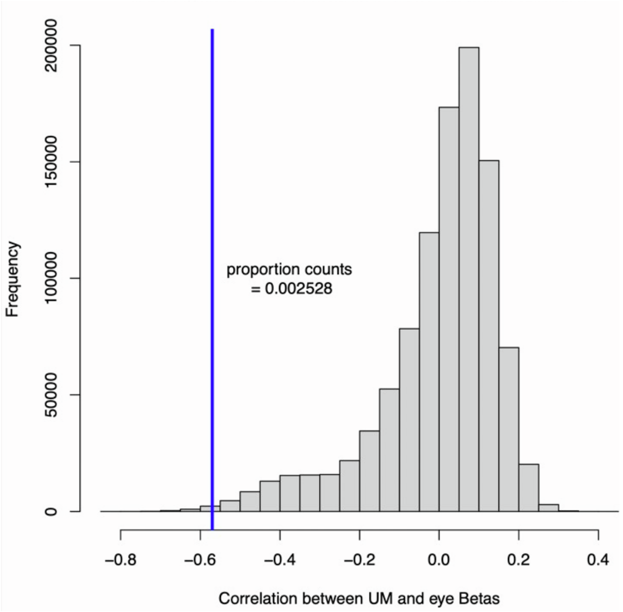

Figure S3. Histogram of permutation test of the correlation between UM and eye pigmentation betas with one million permutations. Blue line denotes the result of our correlation analysis at -0.57.

Study	Nominal hit; gene	P-value	Beta	Replication P-value	Replication Effect
Wills; 2024	22:44887606	9.33e-07	2.507	0.1581	0.2792
Wills; 2024	13:42480082	2.60e-06	1.625	0.946	0.0107
Wills; 2024	6:30760948; HCG20	2.77e-06	-0.418	0.0006397	-0.1492
Wills; 2024	2:34322933	3.16e-06	0.857	0.5344	0.0479
Wills; 2024	3:82245336	3.86e-06	-0.419	0.1604	0.0838
Wills; 2024	14:47080040	4.01e-06	1.90	0.9805	-0.0063
Wills; 2024	7:21497458	4.83e-06	1.559	0.4819	-0.1036
Wills; 2024	2:109451118	5.03e-06	2.838	Nearest SNP: 0.9239	0.0090
Wills; 2024	5:40906076	5.09e-06	0.430	0.3948	-0.0714
Wills; 2024	10:60881854	5.83e-06	0.926	0.09327	0.1811
Wills; 2024	5:159364998	5.88e-06	0.552	0.07357	0.0968
Wills; 2024	22:19256751	6.45e-06	1.176	0.4815	-0.0941
Wills; 2024	16:47091730	6.74e-06	1.97	Nearest SNP: 0.9211	0.0268
Wills; 2024	2:14657623	7.72e-06	0.460	0.4794	-0.0319
Wills; 2024	5:156690123	7.79e-06	-0.563	0.7277	0.0304
Wills; 2024	19:31580941	7.81e-06	2.28	0.1281	0.4134
Wills; 2024	20:17863905	8.05e-06	-0.598	0.8991	0.0098

Table S1. Nominally significant hits in Wills Eye Hospital GWAS ($P < 10^{-6}$) and replication beta and p-value in meta-analysis with Wills Eye Hospital summary statistics excluded.

**Hit**	**Gene**	**AF in Europeans**	**AF in African populations**	**If in coding region**
15:28365618	*HERC2*	0.707	0.12	Intron Variant
6:396321	*IRF4*	0.153001	0.0240	Intron Variant
5:1325590	*CLPTM1L*	0.404	0.438	Intron Variant
3:49771990	*IP6K1*	0.166	0.325	Intron Variant
13:21440496	*XPO4*	0.459	0.139	Intron Variant
3:14387519	*RP11-536I6.1*	0.119	0.053	No
6:7136772	*RREB1*	0.755	0.569	Intron Variant

Table S2. Genome-wide significant hits identified in meta-analysis, with allele frequencies in both European and African populations from the 1000 genomes, and the gene consequence as reported by NCBI.

**Study**	**Nominal hit; gene**	**P-value**	**Beta**	**Replication P-value**	**Replication Beta**
Thomsen et al., 2020	3:14385116; *RP11-536I6.1*	2.18e-06	0.16	7.179e-06	0.3456
Thomsen et al., 2020	6:7080823; *RREB1*	2.37e-06	-0.3237	0.00077	-0.2023

Table S3. Two nominal hits labeled by target study, chromosome and base pair, nearest gene, p-value from target GWAS, effect size (beta) in summary statistics, and then replication p-value and beta from meta-analysis of the other three studies excluding the one in which the nominal hit was identified.

Study	Nominal hit; gene	P-value	Beta	Replication P-value	Replication beta
Mobuchon et al., 2022	2:134821253	6.30E-06	0.412	0.0518	-0.0211
Mobuchon et al., 2022	5:114032425	1.62E-06	-0.430	0.0473	-0.0429
Mobuchon et al., 2022	9:100900782; *CORO2A*	3.74E-06	0.385	0.5016	0.0596
Mobuchon et al., 2022	10:86358506	1.02E-06	0.418	0.1683	-0.0653
Mobuchon et al., 2022	11:119923914; *ENSG00000255216*	7.61E-06	-1.83	0.8525	-0.0564
Mobuchon et al., 2022	13:59970348	6.00E-06	1.33	0.2087	0.2630
Mobuchon et al., 2022	18:65497221; *DSEL-AS1*	5.23E-06	0.924	0.1298	-0.2965
Mobuchon et al., 2022	X:22574497; *PTCHD1-AS*	4.85E-06	0.548	NA	NA
Thomsen et al., 2020	2:141722127; *LRP1B*	5.98E-07	0.316	0.060	0.168
Thomsen et al., 2020	2:223550985; *MOGAT1*	9.08E-06	0.319	0.842	0.0116
Thomsen et al., 2020	4:157116926	2.04E-06	-0.416	0.269	0.0818
Thomsen et al., 2020	10:117947419; *GFRA1*	8.97E-06	-0.36	0.1202	-0.0780
Thomsen et al., 2020	12:100628758; *DEPDC4*	9.18E-06	-0.36	0.6533	-0.0292
Thomsen et al., 2020	14:90421558	1.42E-07	0.57	0.167	0.1353
Thomsen et al., 2020	15:52467546; *GNB5*	4.24E-06	0.314	0.9227	0.0060
Thomsen et al., 2020	17:62008232; *CD79B*	9.83E-06	0.277	0.1034	-0.0824
Thomsen et al., 2020	18:36048557; *ENSG00000305237*	1.21E-05	-0.56	0.9216	-0.0088
FinnGen	1:112429807; *KCND3*	7.85E-06	-0.54	0.7071	-0.0480
FinnGen	2:85983820; *ATOH8*	4.76E-06	0.359	0.8092	0.0198
FinnGen	6:38766488; *DNAH8*	7.36E-06	0.36	0.7174	0.0157
FinnGen	8:119556112; *SAMD12*	3.57E-06	1.37	0.1464	0.3075
FinnGen	8:31981670; *NRG1*	5.51E-06	0.389	0.2414	0.0594
FinnGen	11:120732349; *GRIK4*	3.26E-06	-0.566	0.2624	-0.0622
FinnGen	16:5839224; *RBFOX1*	7.87E-06	0.44	0.2581	0.0596
FinnGen	22:23395411	6.35E-06	-0.40	0.6223	-0.0198

Table S4. Nominally significant hits in the Thomsen et al, Mobuchon et al, and FinnGen ($P < 10^{-6}$) that did not replicate in the GWAS and replication beta and p-value in meta-analysis with respective summary statistics excluded.
